# Supplementary material for: RiboDiffusion: tertiary structure-based RNA inverse folding with generative diffusion models
Source: Bioinformatics. 2024 Jun 28;40(Suppl 1):i347–56. doi: 10.1093/bioinformatics/btae259 (PMC11211841; doi:10.1093/bioinformatics/btae259)
Supplement: btae259_Supplementary_Data [file btae259_supplementary_data.pdf]

RiboDiffusion: Tertiary Structure-based RNA Inverse Folding with Generative Diffusion Models

Han Huang,[1,2,†] Ziqian Lin,[1,3,†] Dongchen He,[1] Liang Hong[1] and Yu Li[1,*]

[1]Department of Computer Science and Engineering, CUHK, Hong Kong SAR, China, [2]School of Computer Science and Engineering, Beihang University, Beijing, China and [3]Nanjing University, Nanjing, China

A. Dataset

Our dataset has $7,322$ experimentally determined RNA 3D structures. We provide the length histogram of these structures in Figure 1.

B. Experiment Details and Results

B.1. Secondary Structure-based Methods

We apply several RNA secondary structure-based inverse folding and protein inverse folding methods to compare model performance. For RNA secondary structure inverse folding methods, we extract secondary structures in dot-bracket form through DSSR (Lu et al., 2015). We use the default optimized hyperparameters of these methods. For LEARNA and MetaLEARNA (Runge et al., 2019), we set the design time limit to 600 seconds.

B.2. Protein Inverse Folding Methods

GVP-GNN (Jing et al., 2021), PiFold (Gao et al., 2023) and StructGNN (Ingraham et al., 2019) are models based on graph neural networks which are first used for protein inverse folding methods. These methods can well extract geometric features using their graph neural network module. As a result, we construct RNA geometric features as model input.

For GVP-GNN, we use the same input features of RiboDiffusion as RiboDiffusion has a structure module based on GVP-GNN. The scalar node features contain dihedral angles of each nucleotide. The vector node features consist of forward and reverse vectors of sequential C1' atoms, as well as the local orientation vectors of C1' to C4' and N1/N9. The initial embedding of each edge consists of its connected C1' atom's direction vector, Gaussian radial basis encoding for their Euclidean distance, and sinusoidal position encoding of the relative distance in the sequence.

StructGNN consists of two parallel encoders to obtain embeddings of substructures and molecules, followed by a feed-forward neural network for prediction. PiFold contains PiGNN layers considering multi-scale residue interactions in node, edge, and global context levels of the graph and a linear layer. For PiFold and StructGNN, we construct distance, angle, and direction features for single or paired nucleotides similar to those in protein. The scalar node features contain dihedral angles of each nucleotide and Gaussian radial basis encoding for every atom pair among C4', C1', N1/N9 of each nucleotide. The vector node features consist of the local orientation vectors of C1' to C4' and N1/N9. The scalar edge features contain Gaussian radial basis encoding of every atom pair among C4', C1', N1/N9 of two different nucleotides, as well as quaternions of relative rotation between their local coordinate systems. The vector edge features consist of the orientation vectors of C1' of one nucleotide to C4' and N1/N9 in a different nucleotide. In these features, C4', C1', N1/N9 in nucleotide correspond to N, C_α, C in protein residues.

Protein inverse folding methods exploit the geometric features of protein molecules. By constructing similar geometric features in our 3-atom RNA backbones, we can retrain these models on the RNA dataset. As a result, these methods can be applied to the RNA inverse folding problem.

B.3. Metric

Recovery rate. This metric evaluates the quality of inverse folding from the perspective of sequence similarity. It is not perfect because it cannot directly characterize the possibility of sequences folding into a specified structure, but it still has a certain reference value. We plot the random mutation ratio versus the free energy of the sequence folding into a given secondary structure (extracted from the tertiary structure) in Figure 2. Folding into the structure is more likely when the recovery rate is relatively high. Moreover, our method has lower free energy than random mutation at the same recovery rate.

F1 Score for secondary structure alignment. F1 Score is defined between the secondary structure of the generated sequence predicted by RNAfold (Gruber et al., 2008) and the secondary structure extracted from the input tertiary structure. This metric reflects whether the generated sequence satisfies the folding constraints from the secondary structure level. However, since the secondary structures derived from both methods may have errors, we remove data that may have large errors based on the F1 score of the native sequence with a threshold of 0.7.

Rfam success rate. We use Rfam's covariance model to evaluate whether the sequence obtained by inverse folding maintains the same family information with the original RNA. Sequences within the curated family are generally considered to have conserved structures and similar functions. It is also of significance to discover such new sequences through inverse folding. Specifically, we define the success case as whether the bit score of the generated sequence is larger than the gathering threshold.

B.4. Hyperparameters

Here we list the main hyperparameters we used in our model. We construct nucleotide graphs with top-10 neighbors and stack 4 layers for the graph neural network in the structure module, where the node feature dimension is 512 and the edge feature dimension

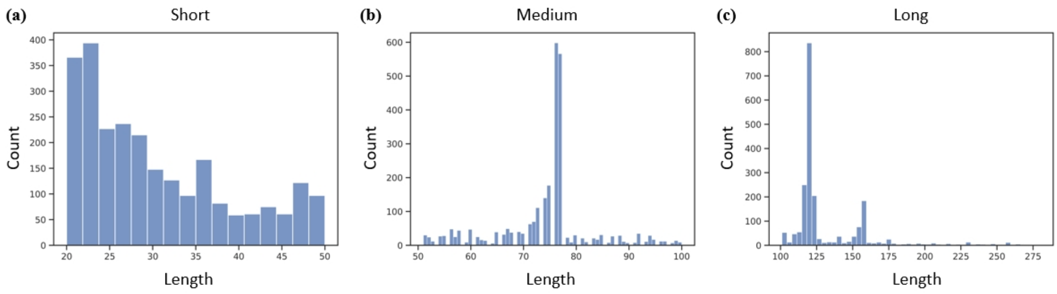

Fig. 1: **Length distribution of the experimentally determined structures. (a)-(c)** Length distribution of short ($L <= 50$nt), medium ($50\text{nt} < L <= 100$nt) and long ($L > 100$nt) RNA.

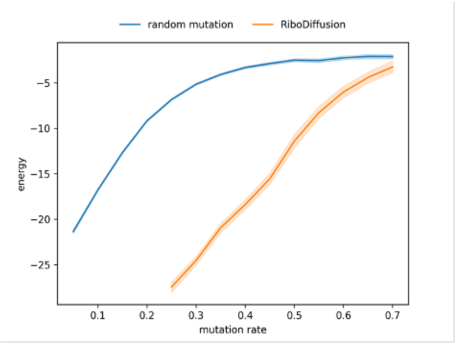

Fig. 2: **The correlation between different mutation rates and free energy** (with random mutation and RiboDiffusion).

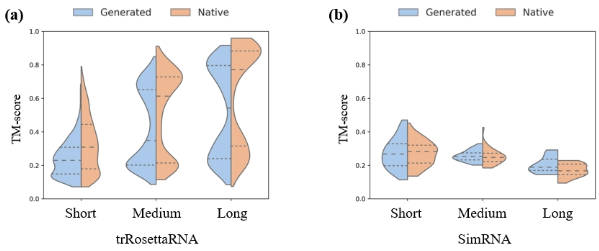

Fig. 3: **In-silico folding validation results of trRosettaRNA and simRNA.** In-silico folding validation results that show the TM-score between structures predicted by trRosettaRNA or simRNA and the given fixed RNA backbones (on *Seq. 0.4* split). *Native* represents structures predicted from original sequences of given backbones as references, while *Generated* represents structures predicted from generated sequences.

is 128. For the sequence module consisting of 8 blocks, we keep the 512 dimensions and use 8 attention heads. Our model is trained 40 epochs with the learning rate 0.0002.

B.5. Extra In-silico Tertiary Structure Folding Results

To alleviate concerns about the independence of structure prediction tool and inverse folding models, we use two extra computational tools, trRosettaRNA (Wang et al., 2023) and SimRNA (Boniecki et al., 2016), to obtain tertiary structures of generated RNA sequences. We also use these tools to predict tertiary structures from the original native sequences. As depicted in Figure 3(a), generated and native sequences have similar TM-score distribution when predicted by trRosettaRNA. The result of SimRNA is shown in Figure 3(b). The performance of SimRNA is relatively poor, which indicates that although generated sequences have a similar TM-score distribution to natural sequences, the refolding evaluation based on SimRNA may have a large error and uncertainty.

Besides RhoFold (Shen et al., 2022), we also provide 3D visualized results of DRFold (Li et al., 2023) and trRosettaRNA, which are shown in Figure 6.

B.6. Results on New RNA Structures

We evaluate the newly published RNA structures between 2023 and 2024 as an additional reference for our model. After removing redundancy and removing RNAs similar to the training set, we present 8 structures that have not been trained by RiboDiffusion and RhoFold. The result is displayed in Table 3.

B.7. Performance on CASP15

To assess the generalizability of the model, RiboDiffusion is tested on six natural RNAs in CASP15 without any overlap with the training set. As shown in Figure 4 (a) and (b), the performance of RiboDiffusion in complex RNA backbone structures is impressive, which is demonstrated by an average recovery rate of 0.56. Furthermore, the TM-score values of generated sequences are similar to the native sequences. However, it is important to note that the results of in-silico folding on CASP15 need more follow-up validation, as the TM-score value used as a reference is not satisfactory.

B.8. Ablation Studies

We perform additional ablation studies to validate the necessity of the sequence module and the improvement of data augmentation. We train the models in a sequence similarity split and a structure similarity split and report the results in Table 1. In our diffusion model formulation, adding the sequence module facilitates performance improvement.

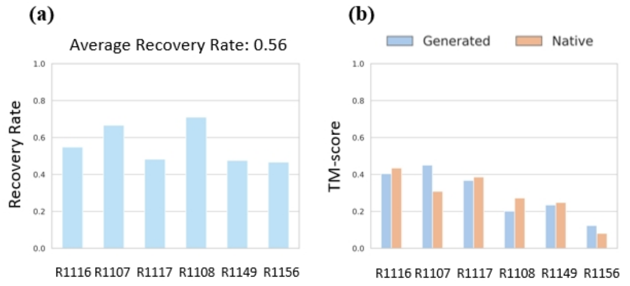

Fig. 4: **Performance on CASP15.(a)** A bar chart shows the recovery rate of RiboDiffusion on six natural RNAs in CASP15. **(b)** A bar chart displays the TM-score between predicted structures of RiboDiffusion-generated sequences and given RNA backbones. The TM-score of predicted structures from native sequences is displayed as a reference.

Table 1. Mean recovery rate (%) on ablation studies.

Method	*Seq.*	*Struct.*
RiboDiffusion	58.96	66.40
RiboDiffusion w/o seq	57.82	64.26

B.9. Running Time and Scalability Analysis.

The inference time of diffusion-based models is largely dependent on the number of steps in the sampling process. For the run-time analysis, we use 50 steps identical to those in our other experiments. On a GeForce RTX 3090 GPU, we report wall clock times of RiboDiffusion generation with different lengths of RNA and different numbers of sequences generated simultaneously in Figure 5. RiboDiffusion can finish the inverse folding of 200 nt RNA in just one second when generating a sequence. However, when generating 128 sequences simultaneously, RiboDiffusion experiences a significant increase in processing time, leading to limitations in scalability. We believe that the running speed of RiboDiffusion can be further improved in the future by accelerating the diffusion models, which is currently an emerging topic in machine learning.

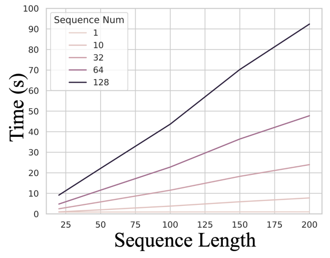

Fig. 5: **Running time and scalability analysis.** A line chart shows the relationship between running time and RNA sequence length when predicting different numbers of RNA sequences simultaneously.

B.10. Extra Results on Secondary Structure Based Methods

We report extra results of secondary structure-based inverse folding methods in Table 2. These methods obtain high F1 scores because they directly use energy optimization to obtain sequences, making it unfair to compare with other methods. It is difficult for secondary structure-based inverse folding methods to generate new sequences in the same family due to the information loss compared to the tertiary structure input, even for tRNA with a more conservative shape.

Table 2. Comparison of secondary structure similarity and success rate of family preservation. The F1 score is an unfair metric for energy-optimized methods.

		rnainverse	MCTS	learna	metalearna
Seq 0.8	F1*	0.990	0.918	0.750	0.905
	Suc.	0.000	0.000	0.000	0.000
Seq 0.6	F1*	0.991	0.922	0.764	0.916
	Suc.	0.000	0.000	0.000	0.000
Seq 0.4	F1*	0.987	0.916	0.796	0.928
	Suc.	0.000	0.000	0.000	0.000
Str 0.6	F1*	0.990	0.915	0.776	0.913
	Suc.	0.000	0.000	0.000	0.000
Str 0.5	F1*	0.985	0.900	0.789	0.919
	Suc.	0.000	0.000	0.000	0.000
Str 0.4	F1*	0.987	0.901	0.762	0.911
	Suc.	0.000	0.000	0.000	0.000

B.11. Results on Remaining Dataset Splits

Extra results of different dataset splits are shown in Figure 7, 8, 9. We show the bivariate distribution of sequence length and recovery rate for RiboDiffusion on test set splits including *Seq. 0.6*, *Seq. 0.8*, *Struct. 0.5* and *Struct. 0.6* in Figure 7. We provide additional violin plots displaying the TM-score performance of RiboDiffusion-RhoFold pipeline about RNA length on test set splits including *Seq. 0.6*, *Seq. 0.8*, *Struct. 0.5* and *Struct. 0.6* in Figure 8. In Figure 9, four different types of RNA are tested to evaluate the performance of RiboDiffusion. The results show that RiboDiffusion performs better on tRNA compared to rRNA. However, its performance in sRNA and ribozyme may be limited due to the scale of the relevant training data.

References

M. J. Boniecki, G. Lach, W. K. Dawson, K. Tomala, P. Lukasz, T. Soltysinski, K. M. Rother, and J. M. Bujnicki. Simrna: a coarse-grained method for rna folding simulations and 3d structure prediction. *Nucleic acids research*, 44(7):e63–e63, 2016.

Z. Gao, C. Tan, and S. Z. Li. Pifold: Toward effective and efficient protein inverse folding. In *ICLR*, 2023.

A. R. Gruber, R. Lorenz, S. H. Bernhart, R. Neuböck, and I. L. Hofacker. The vienna rna websuite. *Nucleic acids research*, 36 (suppl_2):W70–W74, 2008.

J. Ingraham, V. Garg, R. Barzilay, and T. Jaakkola. Generative models for graph-based protein design. *NeurIPS*, 32, 2019.

B. Jing, S. Eismann, P. Suriana, R. J. L. Townshend, and R. Dror. Learning from protein structure with geometric vector perceptrons. In *ICLR*, 2021.

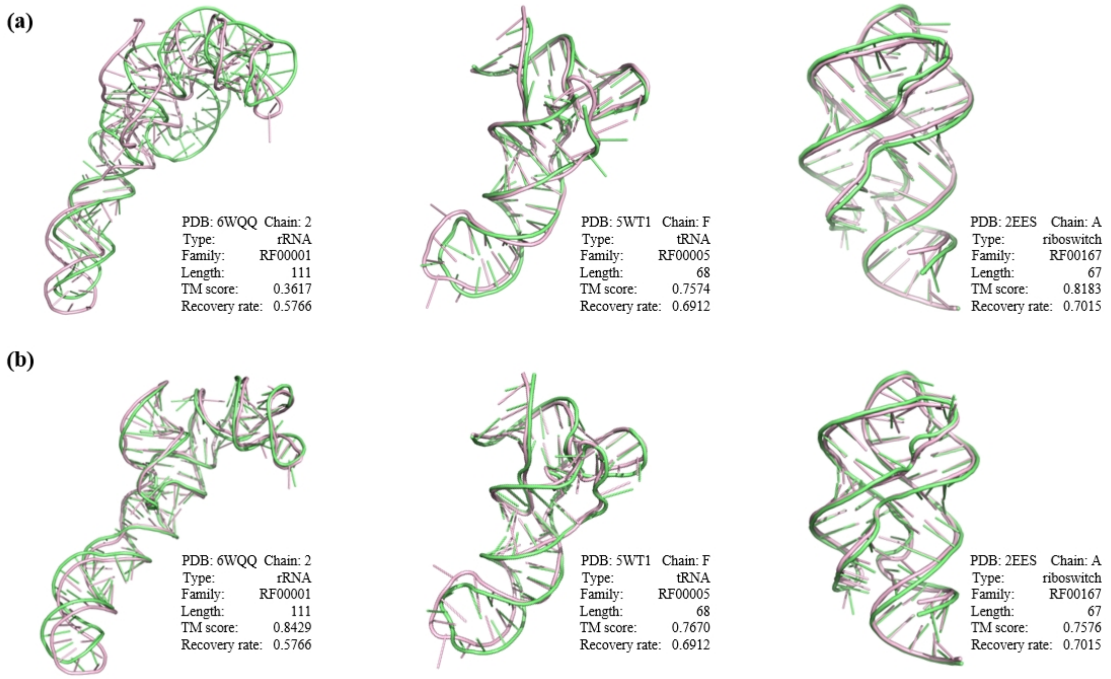

Fig. 6: **In-silico folding visualized results of DRFold and trRosettaRNA .(a)** Visualization of input RNA structures (pink) and predicted structures (green) of RiboDiffusion-DRFold pipeline. **(b)** Visualization of input RNA structures (pink) and predicted structures (green) of RiboDiffusion-trRosettaRNA pipeline.

Table 3. Results on newly published RNA structures. TM-score (generated) is calculated between the given structure and the refolded structure from the RiboDiffusion-RhoFold pipeline. TM-score (native) is calculated between the given structure and the predicted structure of RhoFold with the original native sequence.

PDB id	Recovery rate	TM-score (generated)	TM-score (native)	Generated sequence
7wii_V	0.5918	0.4209	0.3573	GGACCGUCCGCCAACAACGCUCCCCGAAAGGGGAGCAGCG GGAGGUCCA
7xk1_B	0.5556	0.7460	0.7842	CGGAGGUGGCGCAGUGGUAGCGCAGGCGAGUUCAACUCGC CAGGCGCGGGUUCGAUUCCCGUCCUCCGGCCC
8sh5_R	0.5747	0.2632	0.3472	GCGAAACUGGCAGAAUCGGUUAUGAGUUAGUCGAGCGAGA CACGCUCACCCACCUUUUUAGGUUGGCUAACCGUUCGCUC GUUUUGA
8t2a_R	0.5889	0.3275	0.4005	GGCUGCCGGAGUGCUUGUUGUCGUAGCCGGCAUGGAAAGA CCAUGUGCUCGGCUACCCUUCGGGGUGUGAGCUACGGCAC GACGGUGGUC
8fn2_B	0.6964	0.6011	0.3474	GUCUGGUGGCCAUAGAAUCAAGGAACCACCUGAUCCCAUC CCGAACUCAGAAGUUAAGCUUGAUAUCGGUGAUGAUAUUG CGUUUUCGCGAGAAACUAGCGAACUGUCAGAA
8gxb_B	0.6667	0.1951	0.1607	GAGCGUUGCUCGCAAGCGCCGCAUUGCACUUCGCGGCAGA GGUGUUAAUAAAAAGAAGCG
8ine_5	0.7417	0.9157	0.9529	GGGUACGGCCAUACUUCCCUGAAAACACCGAUUCCCCUCC GAUCAUCGAAGUUAAGCAGGGACAGGCUUGGUUAGUACUC GUGUCGGAGACGAACUGGGAACACCGAGUGCUGUACCCUU
8ipy_8	0.5613	0.2557	0.5929	CAAUUCUCGACUCAGAAUAUUUGGCUUCCUCUUCGUUGAA GAACGCAGCAAAAUGCGAUAAGCGAUAUGAGUUGCAAACA UAAAAGAGUAUUAGGGGUUCGAACGCAAAGGCGCUCCCAG UUGAAAUCUGGGAGUACAGCUCUUUCAGUCUCUUG

Y. Li, C. Zhang, C. Feng, R. Pearce, P. Lydia Freddolino, and Y. Zhang. Integrating end-to-end learning with deep geometrical potentials for ab initio rna structure prediction. *Nature Communications*, 14(1):5745, 2023.

X.-J. Lu, H. J. Bussemaker, and W. K. Olson. Dssr: an integrated software tool for dissecting the spatial structure of rna. *Nucleic acids research*, 43(21):e142–e142, 2015.

F. Runge, D. Stoll, S. Falkner, and F. Hutter. Learning to design rna. In *ICLR*, 2019.

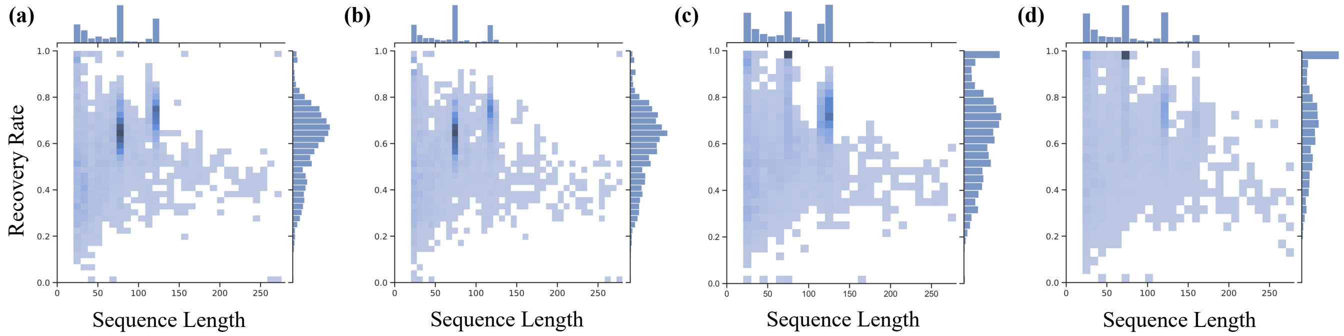

Fig. 7: **Bivariate distribution of sequence length and recovery rate for RiboDiffusion. (a)-(d)** Four joint histplots of the bivariate distribution between sequence length and recovery rate on test set splits including *Seq. 0.6*, *Seq. 0.8*, *Struct. 0.5* and *Struct. 0.6*.

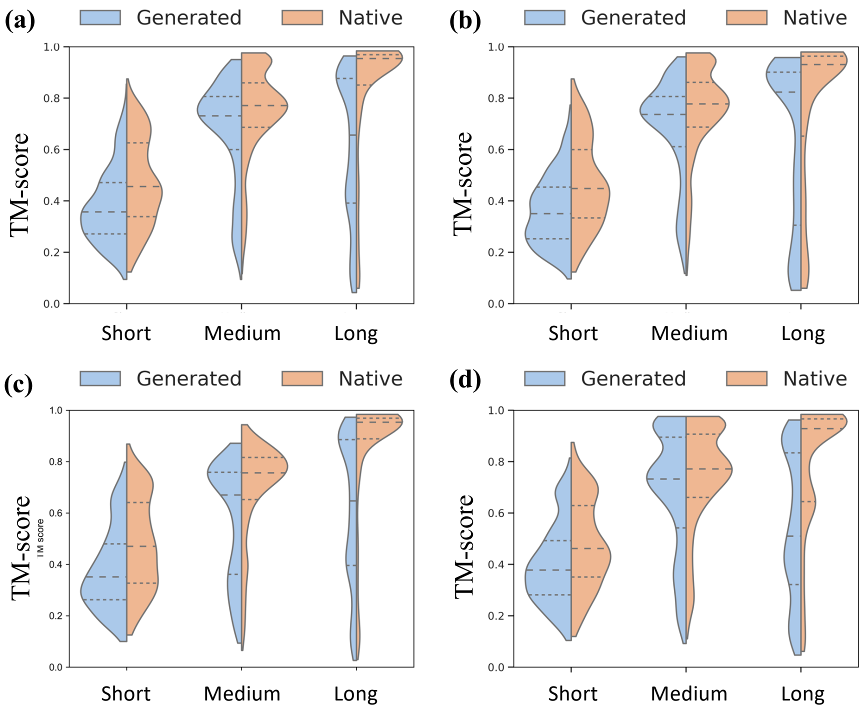

Fig. 8: **TM-score performance of RiboDiffusion-RhoFold pipeline about RNA length. (a)-(d)** Four violin plots compare the TM-score of structures predicted by RhoFold between generated RNA and native RNA on short, medium, and long RNA data in test set splits including *Seq. 0.6*, *Seq. 0.8*, *Struct. 0.5* and *Struct. 0.6*.

T. Shen, Z. Hu, Z. Peng, J. Chen, P. Xiong, L. Hong, L. Zheng, Y. Wang, I. King, S. Wang, et al. E2efold-3d: end-to-end deep learning method for accurate de novo rna 3d structure prediction. *arXiv preprint arXiv:2207.01586*, 2022.

W. Wang, C. Feng, R. Han, Z. Wang, L. Ye, Z. Du, H. Wei, F. Zhang, Z. Peng, and J. Yang. trrosettarna: automated prediction of rna 3d structure with transformer network. *Nature Communications*, 14(1):7266, 2023.

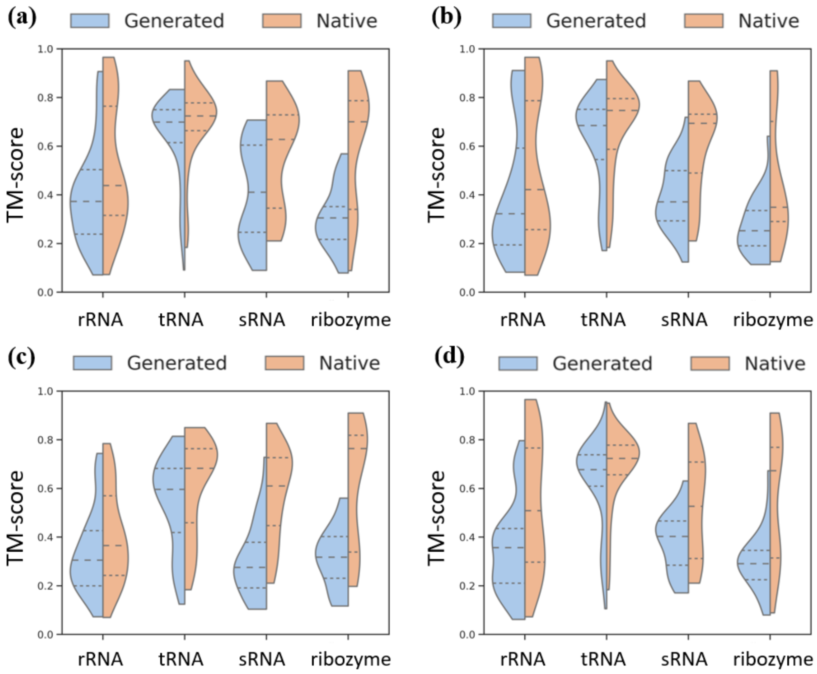

Fig. 9: **TM-score performance of RiboDiffusion-RhoFold pipeline about RNA type. (a)-(d)** Four violin plots compare the TM-score of structures predicted by RhoFold between generated RNA and native RNA on different types of RNA including rRNA, tRNA, sRNA, and ribozyme in test set splits including *Seq. 0.6*, *Seq. 0.8*, *Struct. 0.5* and *Struct. 0.6*.
